# Replacement of saturated fatty acids with linoleic acid in western diet attenuates atherosclerosis in a mouse model with inducible ablation of hepatic LDL receptor

**DOI:** 10.1038/s41598-023-44030-9

**Published:** 2023-10-06

**Authors:** Stephanie D. Burr, Yongbin Chen, Christopher P. Hartley, Xianda Zhao, Jun Liu

**Affiliations:** 1 Department of Biochemistry and Molecular Biology, Mayo Clinic College of Medicine and Science, Rochester, MN 55905 USA; 2 Division of Endocrinology, Diabetes, Metabolism and Nutrition, Mayo Clinic in Rochester, Guggenheim Building 14-11A, 222 3Rd Avenue SW, Rochester, MN 55905 USA; 3 Department of Laboratory Medicine and Pathology, Mayo Clinic in Rochester, Rochester, MN 55905 USA

**Keywords:** Biochemistry, Molecular biology, Physiology

## Abstract

Dietary saturate fatty acids (SFAs) have been consistently linked to atherosclerosis and obesity, both of which are characterized by chronic inflammation and impaired lipid metabolism. In comparison, the effects of linoleic acid (LA), the predominant polyunsaturated fatty acid in the Western diet, seem to diverge. Data from human studies suggest a positive association between high dietary intake of LA and the improvement of cardiovascular risk. However, excessive LA intake has been implicated in the development of obesity. Concerns have also been raised on the potential pro-inflammatory properties of LA metabolites. Herein, by utilizing a mouse model with liver-specific Ldlr knockdown, we directly determined the effects of replacing SFAs with LA in a Western diet on the development of obesity and atherosclerosis. Specifically, mice treated with a Ldlr ASO were placed on a Western diet containing either SFA-rich butter (WD-B) or LA-rich corn oil (WD-CO) for 12 weeks. Despite of showing no changes in body weight gain or adiposity, mice on WD-CO exhibited significantly less atherosclerotic lesions compared to those on WD-B diet. Reduced lesion formation in the WD-CO-fed mice corresponded with a reduction of plasma triglyceride and cholesterol content, especially in VLDL and LDL, and ApoB protein levels. Although it increased expression of proinflammatory cytokines TNF-α and IL-6 in the liver, WD-CO did not appear to affect hepatic injury or damage when compared to WD-B. Collectively, our results indicate that replacing SFAs with LA in a Western diet could reduce the development of atherosclerosis independently of obesity.

## Introduction

Atherosclerosis is the underlying cause for most cardiovascular diseases (CVDs), which together are the leading cause of death in the US and worldwide. During the atherosclerotic development, interplays between the innate immune system with lipid-derived products lead to arterial stiffness, the formation of foam cells, the deposition of plaques of lipids especially cholesteryl ester (CE) and the blockage of the blood vessels^1,2^. Amongst many traditional risk factors for CVDs, obesity is well known for accelerating atherosclerosis by many mechanisms such as increased low-grade, systemic inflammation and abnormal lipid metabolism^3^.

Dietary fatty acids (FAs) play major roles in the development of atherosclerosis. In general, diets rich in saturated FAs are considered both obesogenic and proatherogenic as their consumption increases adiposity, circulating low-density lipoprotein (LDL) cholesterol and inflammatory markers^4,5^. While populations with lower saturated fat intake show lower rates of atherosclerosis, reducing saturated fat in randomized controlled trials has reduced the incidence of CVDs^6^. Accordingly, the current American Heart Association (AHA)/American College of Cardiology guideline is to decrease the intake of saturated fat to 5% to 6% of total daily energy (calorie) intake for individuals with elevated LDL-cholesterol concentration^7^. The implementation strategy recommended is to shift food choices from those high in saturated to those high in polyunsaturated and monounsaturated fats^8^.

Polyunsaturated FAs (PUFAs) have been documented since the 1950s to have a weaker but opposite effect on the blood cholesterol compared to saturated FAs (SFAs)^9^. Consequently, PUFA intake has been advocated to help lower blood cholesterol and thereby alleviate the CVD epidemic in the US. One such recommendation was the replacement of SFAs with linoleic acid (LA), the essential omega-6 (or n-6) PUFA^10^. In fact, the AHA and the Academy of Nutrition and Dietetics recommend its intake at 5% to 10% of the energy^11,12^. Consumption and production of LA-rich vegetable oil such as corn oil, sunflower oil and soybean oil as well as margarine have increased steadily since the 1960s. However, there is a body of previous work showing LA to be more obesogenic than SFAs^5,8,13–15^. Concerns have also been raised that n-6 PUFAs could increase CVD risk because of its potential proinflammatory effects and susceptibility to oxidation when incorporated in LDL^16–18^.

Although a recent meta-analysis reported an association of higher LA intake and lower risk of CVDs and CVD mortality^19^, the effect of LA replacing saturated FAs in dietary fat on atherosclerotic susceptibility in obesity has not been directly examined. To this end, animal models of atherosclerosis in diet-induced obesity provide a highly valuable setting as they allow lesion quantification at the end of study. Herein, we aimed to compare the effects of a high intake of saturated vs LA-enriched polyunsaturated fat along with high cholesterol in a mouse model of pharmaceutically induced atherosclerosis^20,21^. Specifically, two high-fat Western diets enriched with either butter (WD-B) or corn oil (WD-CO) were fed to mice that received an antisense oligonucleotide (ASO) targeting LDL receptor (Ldlr) mRNA in the liver of wild-type C57BL/6 mice. Utilizing this system enables the assessment of the current paradigm surrounding dietary LA and whether LA can induce beneficial outcomes with regard to atherosclerosis.

## Methods

### Mice, diets and ASO treatment

6-week-old male wildtype C57BL/6 J mice were purchased from the Jackson Laboratory (Strain #000664). Mice were maintained in the animal facility at the Mayo Clinic in Rochester, Minnesota with food and water available ad libitum. Mice were fed a standard chow diet until 8 weeks of age, at which time they were placed on a Western diet (17 kcal% protein, 43 kcal% carbohydrate, 40 kcal% fat and 0.21 gm% cholesterol). This diet was either enriched with saturated fatty acids derived from butter (WD-B, D12079B) or PUFAs derived from corn oil (WD-CO, D21050712), both of which were prepared by Research Diets. Additional details concerning the diets and FA composition can be found in Table 1. A GalNAc (N-acetyl-galactosamine) conjugated Gen 2.5 ASO was developed by and obtained from Ionis Pharmaceuticals. The ASO was used to knock down Ldlr and induce atherosclerosis due to C57BL/6J mice being resistant to diet-induced atherosclerosis^20,21^. For a total of 12 weeks on Western diets, mice received weekly intraperitoneal (IP) injection of the Ldlr ASO (2.5 mg/kg). The dosage and timeline have been shown to sufficient to induce atherosclerosis with the Ldlr ASO (21). The mice were fasted for 16 h before euthanasia which occurred via isoflurane overdose. All methods and experiments were performed in compliance with National Institutes of Health guidelines, and followed procedures approved by the Mayo Clinic Institutional Animal Care and Use Committee. All studies reported herein were performed in accordance with ARRIVE guidelines (https://arriveguidelines.org).

**Table 1:** Composition of Western diets enriched with butter (WD-B) or corn oil (WD-CO).

	Western diet: milk fat (SFA)		Western diet: corn oil (PUFA)	
	gm%	kcal%	gm%	kcal%
Components				
Protein	19.8	16.9	19.8	16.9
Carbohydrate	50	42.8	50	42.8
Fat	21	40.3	21	40.3
Total		100		100
Cholesterol (g)	2.1		2.1	
Cholesterol (%)	0.21		0.21	
Ingredients				
Corn oil	10	90	210	1890
Milk fat (butter), Anhydrous	200	1800	0	0

Fatty acids	% Butter	% Corn oil		
SFA	66	13		
Palmitic acid (16:0)	28	11		
Stearic acid (18:0)	12	2		
MUFA	26	26		
Oleic acid (18:1)	22	24		
PUFA	3	60		
Linoleic acid (18:2)	2	~ 58		

### Body composition analysis

Lean and fat masses of individual mice were determined by quantitative nuclear magnetic resonance using an EchoMRI analyzer (Houston, TX) and expressed as a function of body weight. Un-anesthetized animals were placed in a plastic tube that was introduced into the EchoMRI instrument. Body composition, comprising fat mass and lean mass, was determined in approximately 90 seconds per animal.

### Metabolic cage analysis

Indirect calorimetry was performed during light and dark cycles to determine the extent to which treatment affected metabolic parameters during conditions of rest (light cycle) and activity (dark cycle). On the day of the experiment, mice were weighed and acclimated overnight. In a subset of eight mice per group, the habitual ambulatory, rearing, and total activity, oxygen consumption (VO_2_), and carbon dioxide production (VCO_2_) of individual mice were monitored over a 24 h period (12 h light/12 h dark) using a Comprehensive Laboratory Animal Monitoring System (CLAMS) equipped with an Oxymax Open Circuit Calorimeter System (Columbus Instruments). The VO_2_ and VCO_2_ values were used to calculate the respiratory exchange ratio (RER) and VO_2_. RER values were used to determine the basal metabolic rate (in kilocalories per kilogram per hour). Data were analyzed using the CLAX software from Columbus Instruments, exported into Excel and plotted in GraphPad.

### Aortic isolation and staining

Before excision, the aorta was cleared of excess blood via flushing with PBS, followed by the removal of surrounding connective tissue. The aorta was excised from the body cavity starting at the heart (focusing on the heart-aorta root junction), continuing to the aortic arch and corresponding innominate arteries, and ending at the iliac bifurcation junction. After removal, the aorta was fixed in 10% formaldehyde. For *en face* analysis of aortic lesions, the aorta was cut longitudinally and pinned open on black wax pan. Aortas were stained with Sudan IV and images were captured for analysis. Image J (NIH) was used to calculate area of the aortic plaques (Sudan IV positive) and total area of the aorta.

### Fast-protein liquid chromatography

Fast-protein liquid chromatography (FPLC) analysis was performed at the Analytic Services Core of Vanderbilt Diabetes Research and Training Center. Specifically, 100 μl of plasma pooled from 6 mice per group was separated on a Superose 6 column (Amersham Pharmacia). Forty 0.5-ml fractions were collected. Cholesterol and triglyceride analyses were performed on each fraction using standard enzymatic assays. Fractions 16–24 contain VLDL; 25–44, LDL and 45–53, HDL. HDL cholesterol was measured with the enzymatic method after precipitation of VLDL and LDL using polyethylene glycol reagent (PEG). From these data LDL cholesterol was calculated using the Friedewald equation, as long as triglyceride levels were below 400 mg/dl.

### Plasma and hepatic metabolite analysis

Colorimetric and fluorometric assays were used to measure metabolites in fasting plasma. Fasting plasma levels of total triglyceride (Thermo Scientific, TR22421), non-esterified fatty acids (Fujifilm Wako, 999-34691), cholesterol (Sigma Aldrich, A12216), and β-hydroxybutyrate (Cayman Chemicals, NC9044966) were measured following the manufacturer’s instructions. All assays utilized a standard curve with a known standard to determine concentration of specific metabolite. Extraction of hepatic triglyceride (TG) have been previously described^22^. In brief, liver tissue was digested in KOH solution at 55 °C for 4 h. Lipids were then extracted from digested tissue via incubation with H_2_O:EtOH (1:1 ratio) solution. Extracted lipids were then used in previously mentioned assays to determine hepatic concentration which were normalized to tissue mass. Hepatic cholesterol was extracted using 1X RIPA buffer (10 mM Tris–Cl pH 8.0, 1 mM EDTA, 0.5 mM EGTA, 1% Triton X-100, 0.1% SDS, and 140 mM NaCl).

### Liver histology

Liver tissue was fixed in 10% formaldehyde and send to the histology core at Mayo Clinic Arizona for paraffin-embedding, sectioning, and immunohistochemistry. Liver sections were stained with H&E and imaged using the Motic Slide Scanner available through the Mayo Clinic Rochester Microscopy and Cell Analysis Core. The severity of steatosis and inflammation was assessed using the rodent histological scoring system for non-alcoholic fatty liver disease, as established by Liang et al.^23^.

### Western blot analysis

Proteins from the liver were extracted with homogenization in NP-40 lysis buffer (1%N NP-40, 50 mM Tris–HCl pH 7.5, 150 mM NaCl, 0.5% SDS, 0.5 mM Na_3_VO_4_, and 100 mM NaF) with proteinase inhibitors (Roche Applied Science). High-abundance plasma proteins were removed by incubating plasma proteins with trichloroacetic acid and then washing repeatedly with cold acetone. Protein expression was examined via SDS-PAGE western blot. Membranes for western blot analysis were cut, focusing on a specific size range, in order to conserve sample use and reduce off target labeling. Proteins investigated were ApoB-48 and ApoB-100 (Millipore, AB742), Ldlr (R&D, AF2255), and β-actin (Sigma, A1978). Images were captured using an ImageQuant LAS 4000 Imager and analyzed with ImageQuant Software.

### mRNA extraction and real-time PCR

Extraction of mRNA from liver tissue was conducting using TRIzol™ according to the protocol provided by the manufacturer (Thermo Scientific, 15596026). In brief, tissue was homogenized in TRIzol™ reagent, treated with chloroform, and then centrifuged. The mRNA was precipitated with isopropanol and then washed with ethanol. Reconstituted mRNA was used to generate cDNA using high-capacity cDNA reverse transcription kit (Thermo Scientific, 4374966). The cDNA was used with Itaq™ Universal SYBRGreen (BioRad, 1725124) on BioRad CFX96 Touch Real-time PCR machine to determine mRNA expression levels. Results for real-time PCR were evaluated using the BioRAD CFX Manager software and the comparative cycle threshold (ΔΔCt) method was employed for analysis. The levels of mRNA were normalized to β-actin and were presented as relative to the WD-B treatment group. qPCR primers utilized in this study are available on request.

### Graphical depiction and statistical analysis

Each graph depicts the mean and SEM for WD-B and WD-CO treatment groups. The individual data points for each group are depicted in each graph for transparency. Statistical analysis was conducted using GraphPad Prism software (version 9.4.0). All statistical tests consisted of a Student’s t-test comparing between WD-B and WD-CO fed mice. Statistical differences indicated by the following: *p < 0.5, **p < 0.1, ***p < 0.001, and ****p < 0.0001.

## Results

### Diets and diet composition

**Figure 1:**
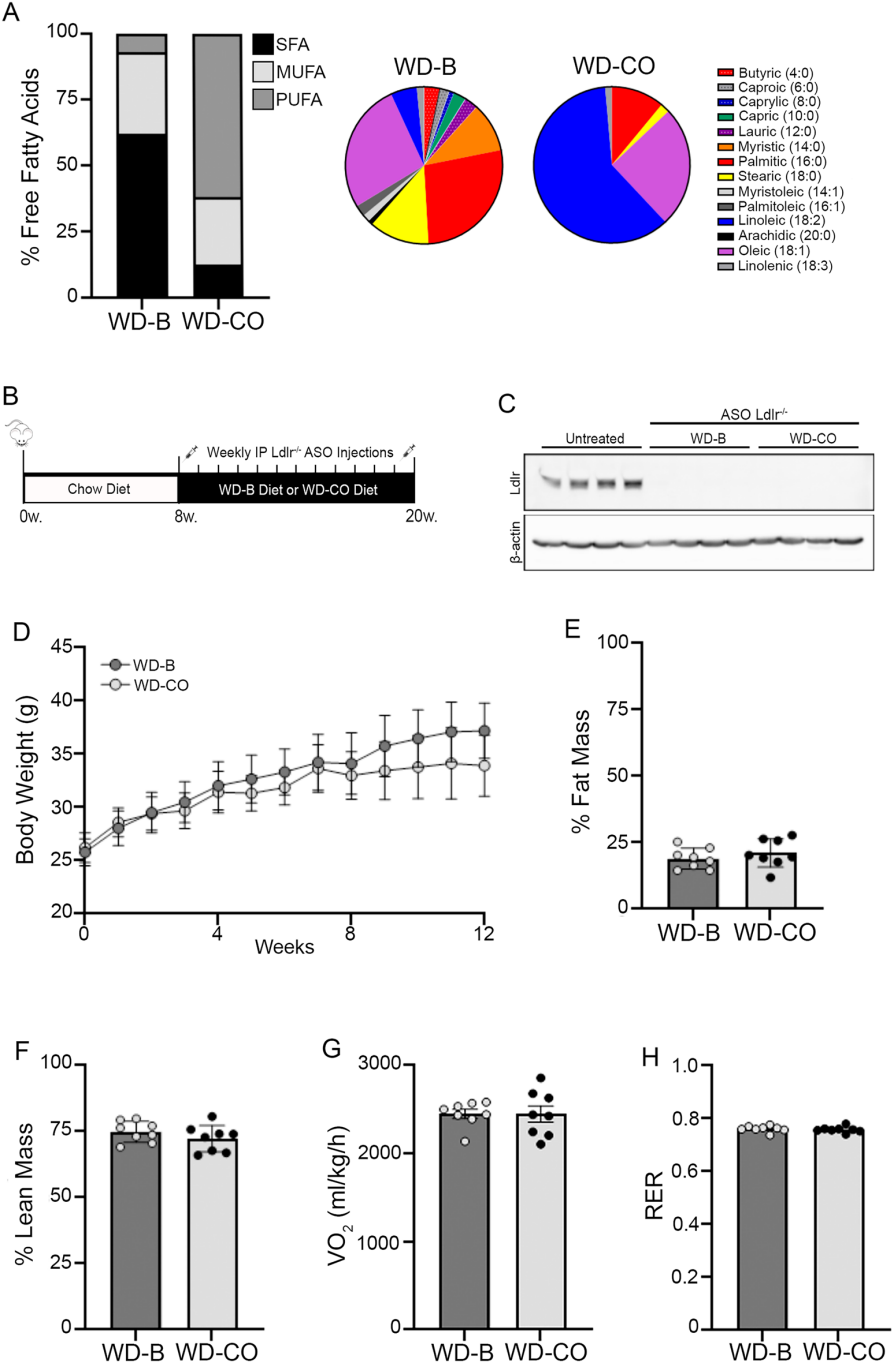
Body weight, body composition and energy expenditure of mice on Western diets. (**A**) the composition of FAs in terms of saturation and specific fatty acid species in WD-B and WD-CO diets. (**B**) experimental timeline and design aimed to induce atherosclerosis via Ldlr^−/−^ ASO (**C**) Western blot for Ldlr expression in liver tissue from untreated and Ldlr ASO treated mice. (**D**) time course of body weight change. (**E**) Percentage of fat mass. (**F**) Percentage of lean mass. (**G**) Whole-body oxygen consumption. (**H**) Whole-body respiratory exchange ratio. Data represented as mean ± SEM with n = 8–10 (**D**–**H**).

As shown in Table 1, both diets contained 16.9% of calorie as protein, 42.8% of calories as carbohydrate, and 40.3% of calories as fat. Per kg of diet, the WD-B contained 200 g of butter and 10 g of corn oil. In comparison, the WD-CO contained 210 g of corn oil and no butter. As a result, the WD-B contained high levels of SFA (62.4%) and low levels of PUFA (6.9%), whereas WD-CO contained low levels of SFA (12.9%) and high levels of PUFA (62%) (Table 1, Supplementary Table S1 & Fig. 1A). Like in the corn oil, the PUFA in WD-CO was primarily linoleic acid (C18:2n-6), with a small amount of linolenic acid (C18:3n-3). The two diets contained similar amounts of MUFA (30.7% vs. 25.1%) and same amount of cholesterol (0.21 gm%).

### Body weight, adiposity and energy metabolism

8-week-old male mice were fed either WD-B or WD-CO for 12 weeks. Concurrently, the mice were treated weekly with intraperitoneal injections of an ASO specifically targeting Ldlr in the liver (Fig. 1B). Previous study showed no sex difference in atherosclerosis induced by Ldlr-ASO, although males tended to have higher plasma cholesterol levels than females^20,21^. At the end of the study, knockdown of hepatic Ldlr expression was confirmed by Western blotting analysis (Fig. 1C). Over the 12-week treatment period, the WD-B-fed mice exhibited similar body weight gain as mice on WD-CO (Fig. 1D). Similarly, the fat and lean mass composition was not different between the two diet groups (Fig. 1E, F). The metabolic cage study showed essentially the same whole-body oxygen consumption rate (VO_2_) (Fig. 1G) and respiratory exchange ratio (RER) (Fig. 1H), indicating no effects of different dietary FAs on energy expenditure or substrate preference.

### Atherosclerosis

**Figure 2:**
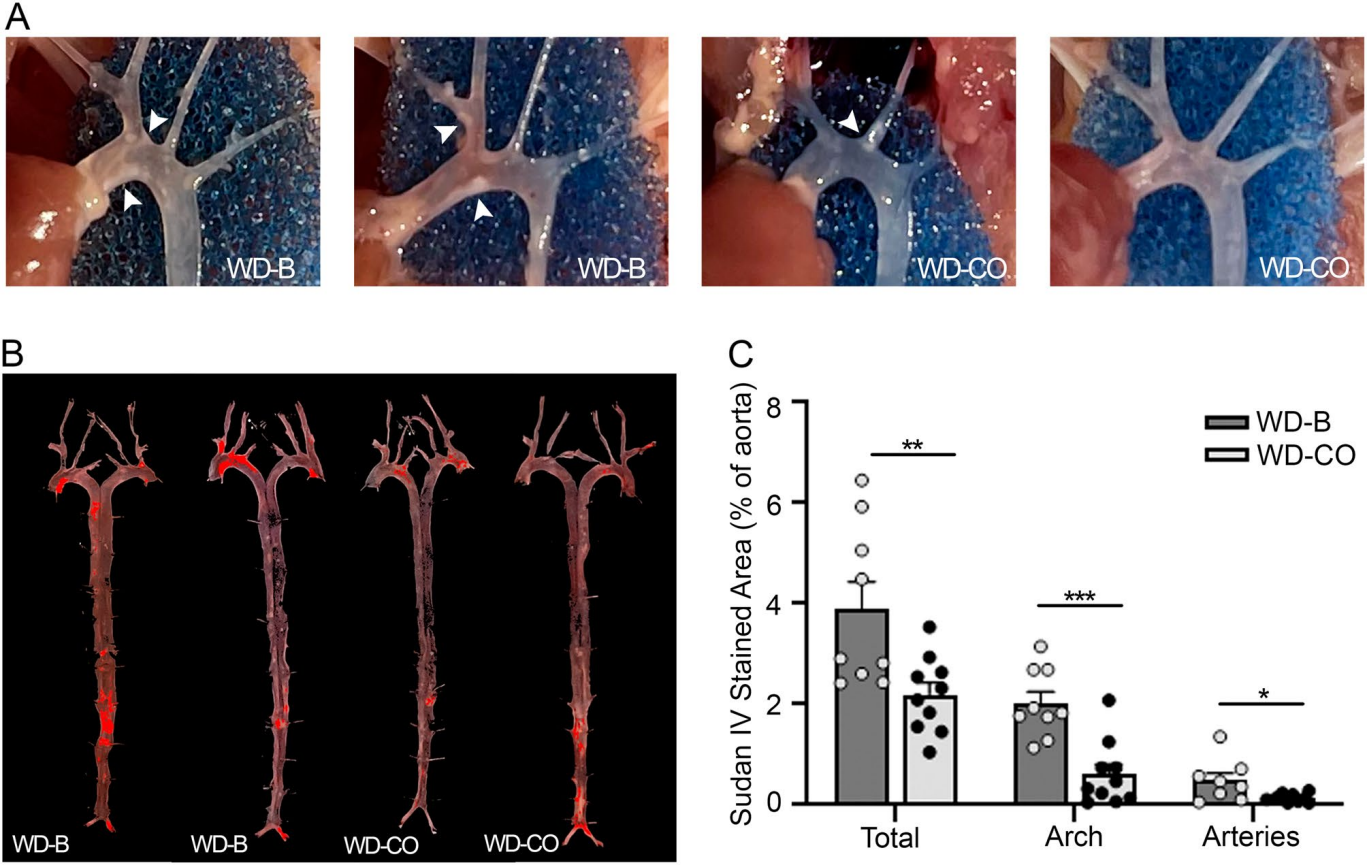
The impact of WD-B and WD-CO on atherosclerotic development in Ldlr ASO mice. (**A**) Representative images depicting plaque formation (white arrow heads) in aortic arch. (**B**) Representative images of en face aorta stained with Sudan IV (bright red) which denoted atherosclerotic lesions. (**C**) Percentage of lesion area over total aortic area (total), aortic arch (arch) and innominate artery (arteries). Data represented as mean ± SEM with n = 8–10 (**C**). Statistical significance was determined by Student’s *t*-test (*p < 0.05, **p < 0.01, and ***p < 0.001).

Direct visualization of aortic arch revealed that sclerotic lesions were prominent in mice from the WD-B diet group. Remarkably, mice in the WD-CO group showed a visible decline in the size of lesions (Fig. 2A, white arrow heads). Both visually and quantitatively, the lesion areas in both aortic arch and innominate artery as stained by Sudan IV were significantly reduced in mice of the WD-CO group, compared with those of the WD-B group (Fig. 2B, C). These results suggest that when SFAs in the Western diet are replaced with the polyunsaturated LA, the hepatic Ldlr-ASO is less effective in inducing atherosclerotic development. This difference appears to be independent of adiposity and diet-induced obesity.

### Plasma lipid and lipoprotein profiles

**Figure 3:**
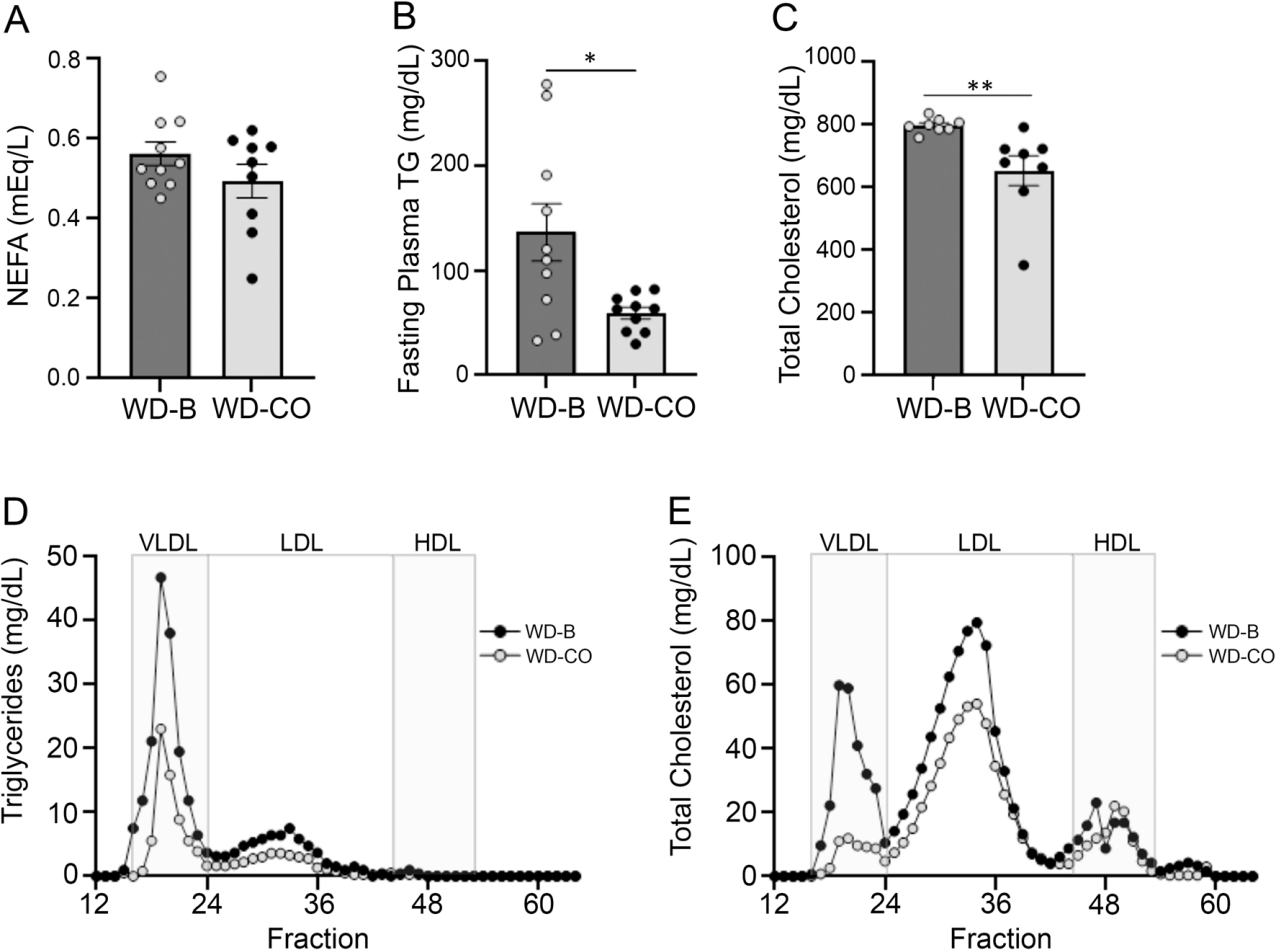
Plasma lipid and lipoprotein profiles of Ldlr ASO mice on WD-B and WD-CO. (**A**) Fasting plasma free FA levels. (**B**) Fasting plasma triglyceride levels. (**C**) Fasting total plasma cholesterol concentration. (**D**) TG concentration in VLDL, LDL, and HDL fractions. (**E**) Cholesterol concentration in VLDL, LDL, and HDL fractions. Data represented mean ± SEM with n = 8–10 (**A**–**C**) and n = 6 pooled samples for FPLC fractionation (**D**, **E**). Statistical analysis consisted of Student’s *t*-test (*p < 0.05 and **p < 0.01).

Fasting plasma lipids were measured in mice treated with WD-B and WD-CO. As shown in Fig. 3A, the plasma free FA levels were not different in mice between the two diet groups. However, the plasma triglyceride (TG) levels in the WD-CO-treated mice were significantly lower than mice in the WB-B group (Fig. 3B). Total plasma cholesterol, which encompasses both free and esterified cholesterol, was found to be significantly lower in the WD-CO mice as well (Fig. 3C). In addition, plasma lipoproteins were fractionated by FPLC and analyzed for cholesterol and triglyceride content. Compared to the mice in the WD-B group, WD-CO-treated mice exhibited decreased levels of cholesterol and TG in both VLDL and LDL fractions (Fig. 3D, E), with VLDL showing the most drastic reductions. While the VLDL-TG was decreased by 26%, the VLDL-cholesterol showed a fourfold decrease in mice of the WD-CO group. These results suggest that the dietary LA exerts a more profound impact on VLDL-cholesterol than VLDL-TG content.

### Liver lipids, ApoB and FA oxidation

**Figure 4:**
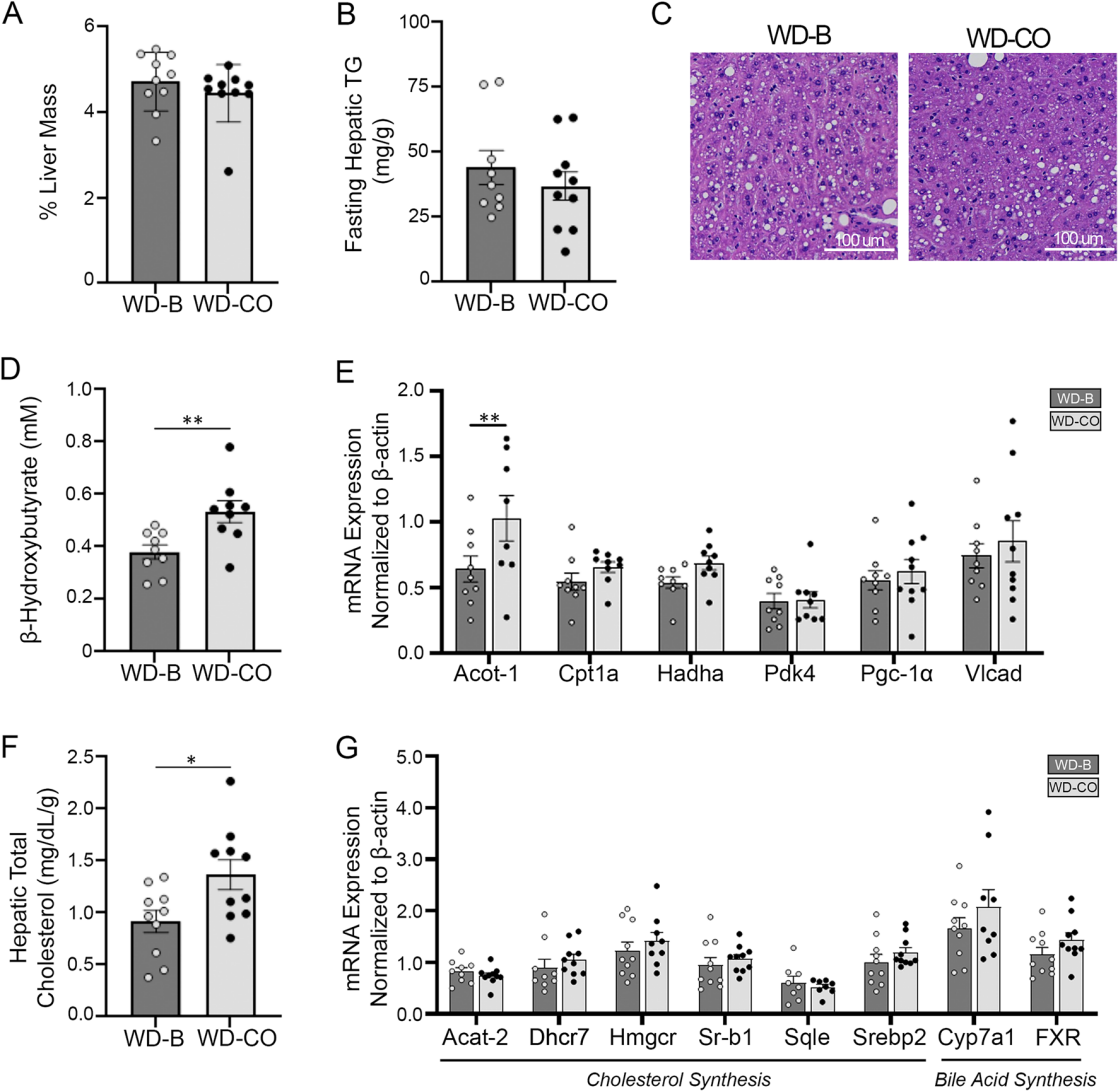
Liver mass, lipid composition, and FA oxidation in Ldlr ASO mice on WD-B and WD-CO. (**A**) Percentage of liver mass compared to body mass. (**B**) Fasting TG levels in liver tissue. (**C**) Representative images of H&E-stained liver sections. (**D**) Fasting plasma concentration of β-hydroxybutyrate. (**E**) Hepatic mRNA expression of FA oxidative associated genes by qPCR. (**F**) Hepatic total (free and esterified) cholesterol levels. (**G**) Hepatic mRNA expression of cholesterol and bile metabolism associated genes by qPCR. Data represented as mean ± SEM with n = 8–10 (**A**–**B**, **C**–**G**). Statistical significance was determined by Student’s *t*-test (*p < 0.05 and **p < 0.01).

Liver mass and hepatic TG content were shown to be similar between WD-B and WD-CO-treated mice (Fig. 4A, B). There was also no difference in the development of hepatic steatosis as revealed by H&E staining (Fig. 4C). Further examination determined that the plasma level of the ketone body β-hydroxybutyrate was significantly higher in the WD-CO fed mice compared to the WD-B fed mice (Fig. 4D), indicating an increased hepatic FA oxidation induced by dietary LA. However, most of the FA oxidation-related genes were expressed at similar levels except for ACOT1, which showed a significantly increased expression in the WD-CO group (Fig. 4E).

**Figure 5:**
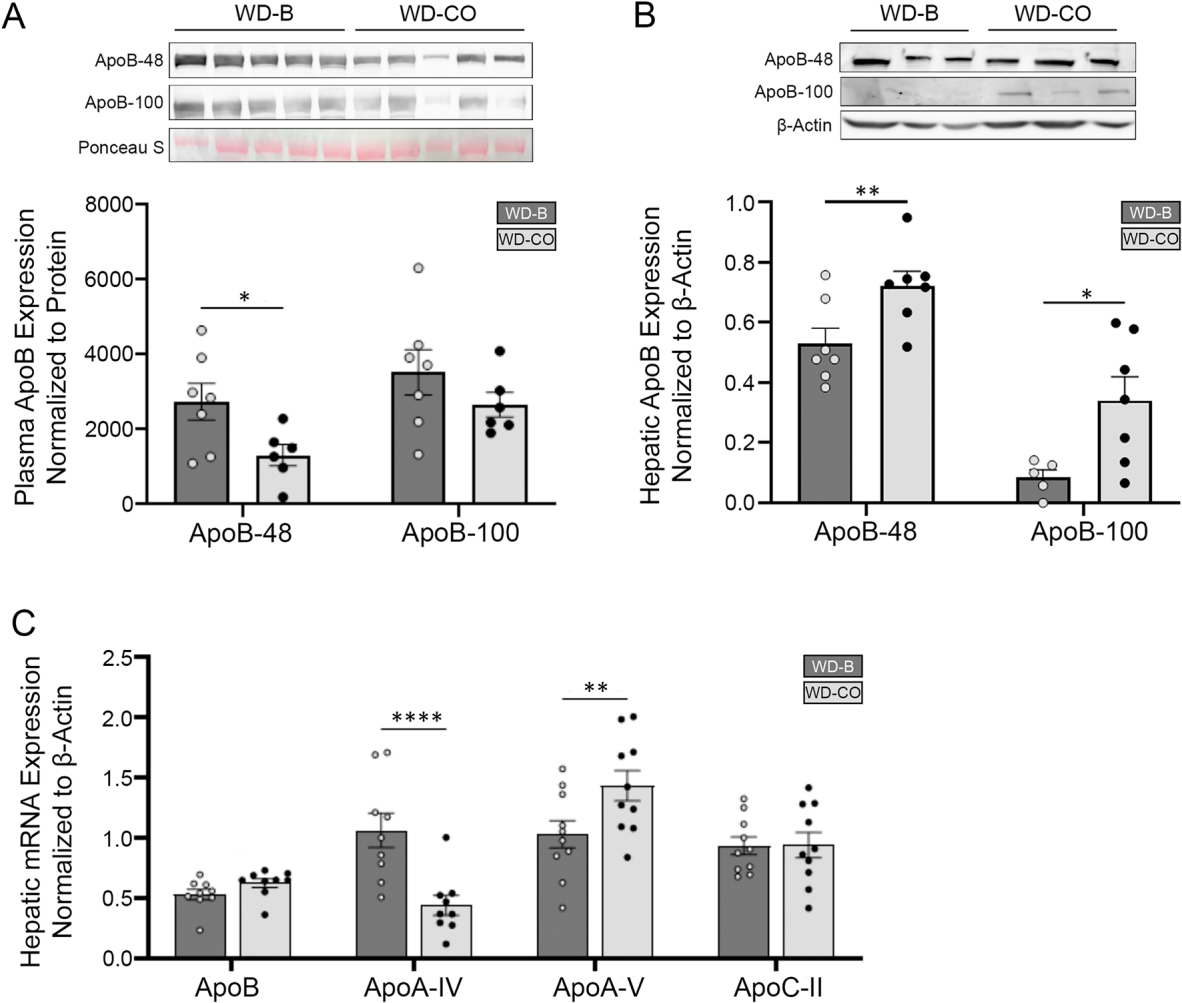
Expression of apolipoproteins in fasting plasma and hepatic tissue. (**A**) Immunoblotting and quantitative analysis of ApoB-48 and ApoB-100 protein in fasting plasma. (**B**) Immunoblotting and quantitative analysis of ApoB-48 and ApoB-100 protein in liver tissue. (**C**) qPCR analysis of hepatic apolipoprotein associated genes. Data represented as mean ± SEM with n = 8–10 (**A**–**C**). Statistical analysis consisted of a Student’s *t*-test (*p < 0.05, **p < 0.01, and ****p ≤ 0.0001).

Interestingly, the hepatic total cholesterol content was significantly higher in mice of the WD-CO group. However, this did not seem to result from altered expression of genes involved in cholesterol or bile acid synthesis (Fig. 4F, G). This correlated with decreased plasma levels of ApoB-48 and ApoB-100 proteins and increased hepatic levels of ApoB-48 and ApoB-100 in these animals (Fig. 5A, B). Interestingly, the mRNA expression of ApoB remained unchanged in the liver (Fig. 5C), suggesting that hepatic ApoB secretion was attenuated in mice of the WD-CO group. Congruently, the hepatic expression profile of apolipoproteins in the WD-CO-fed mice appears to be less favorable for ApoB lipoprotein/VLDL secretion (Fig. 5C). Specifically, expression of ApoA-IV, which is known to promote ApoB lipoprotein secretion ^24,25^, was downregulated in the WD-CO group. ApoA-V, which inhibits VLDL secretion as well as facilitates VLDL-TG hydrolysis^26,27^, was conversely upregulated in these mice. In comparison, the hepatic expression of LPL cofactor ApoC-II remained unaffected.

### Hepatic inflammation and injury

**Figure 6:**
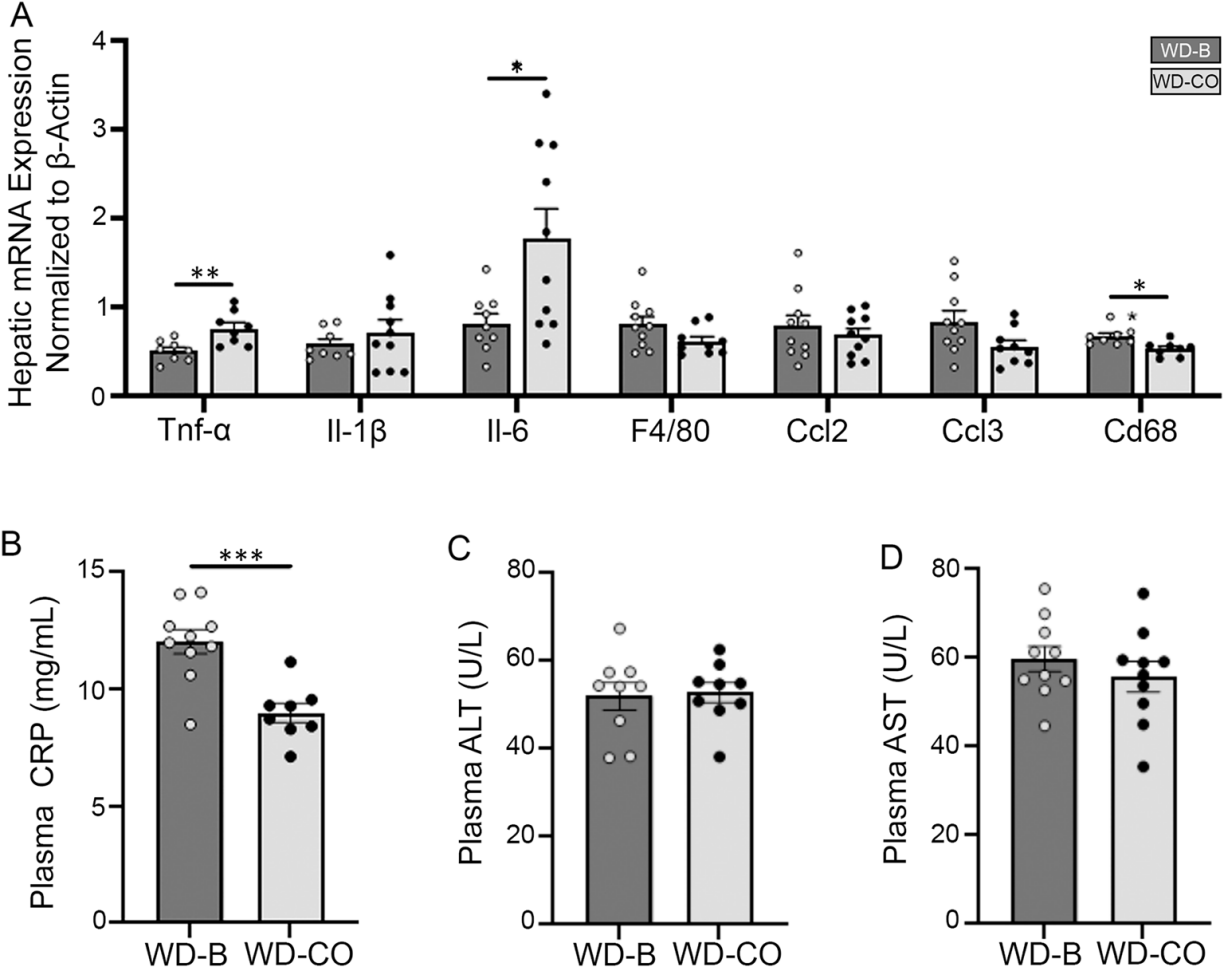
Assessment of hepatic inflammation and injury. (**A**) qPCR analysis of inflammation-associated gene expression in liver. (**B**) Concentration of CRP in fasting plasma. (**C**) Fasting plasma levels of ALT. (**D**) Fasting plasma levels of AST. Data represented as mean + SEM with n = 8–10 (**A**–**C**). Statistical analysis was conducted using a Student’s *t*-test (*p < 0.05 and **p < 0.01, ***p < 0.001).

It was shown previously that chronic treatment with dietary SFA promotes localized hepatic inflammation. Interestingly, the WD-CO treatment caused an increased expression of TNF-α and IL-6 in the liver (Fig. 6A), suggesting that dietary LA may promote hepatic inflammation beyond what is induced by WD-B. However, there appears to be no difference in the liver macrophage recruitment as indicated by the expression of macrophage markers such as F4/80, CCL2, CCL3 and CD68. In addition, pathological review of liver sections found a decreased incidence of microvesicular steatosis in the WD-CO group (Table 2). However, there was no significant difference in either macrovesicular steatosis or inflammation, though both trended lower in the WD-CO group. As reflected by their lower plasma levels of C-reactive protein (CRP), systemic inflammation appeared to be less in mice fed WD-CO diet (Fig. 6B). Furthermore, compared to the WD-B, the WD-CO did not increase the plasma levels of ALT and AST (Fig. 6C, D), suggesting that the dietary LA elicited no further effect on liver damage or injury.

**Table 2:** Histological assessment of liver sections from WD-B and WD-CO fed mice.

	Western diet: milk fat (WD-B)	Western diet: corn oil (WD-CO)	*p* value
Macrovesicular	1 ± 0	0.667 ± 0.167	0.1161
Microvesicular	0.833 ± 0.167	0.167 ± 0.167	0.0474
Hypertrophy	0 ± 0	0 ± 0	–
Inflammation	1.333 ± 0.882	0.333 ± 0.333	0.3486

The liver tissue slides from the mice were independently reviewed by two pathologists, who were blinded to the experimental group assignments.

## Discussion

In the present study, we obtained three significant findings from mice receiving an ASO targeting hepatic Ldlr. Firstly, the substitution of corn oil for butter in the Western diet results in a significant reduction in atherosclerotic development. Secondly, WD-CO diet has lowering effects on plasma lipid and lipoprotein levels. Given LA constituting ~ 98% of all PUFAs in corn oil, these two findings indicate that the dietary LA can contribute to the protection against atherosclerosis under the conditions of impaired hepatic LDL-cholesterol clearance. Thirdly, such a protective effect does not seem to involve altered adiposity or energy metabolism. This is consistent with previous studies that have identified both SFAs and n-6 PUFAs as obesogenic^5,8,13–15^. Thus, our results point to the dietary LA as being beneficial for the cardiovascular health in obesity.

In humans, replacing dietary SFA with omega-6 PUFA was consistently found to lower total blood cholesterol concentrations^28^. It was previously proposed that LA reduces blood cholesterol through the upregulation of LDL receptor and clearance of LDL-cholesterol from plasma. However, this mechanism is unlikely to underlie the plasma cholesterol-lowering effects of LA in our ASO mouse model, of which hepatic Ldlr was knocked down. Compared to the WD-B rich in SFAs, the WD-CO diet rich in LA instead was associated with drastically reduced cholesterol content in the plasma VLDL as well as significantly decreased VLDL-TG. This conceivably would lead to decreased accumulation and deposition of VLDL-cholesterol in macrophages and reduced macrophage-derived foam cell formation. Moreover, compared to those on WD-B, mice on WD-CO showed decreased ApoB protein levels in the plasma and increased ApoB levels in the liver despite of unaltered ApoB mRNA expression. We also observed decreased expression of ApoA-IV, which promote ApoB lipoprotein secretion^24,25^, and increased expression of ApoA-V, which inhibits VLDL secretion^26,27^. Given that cholesterol is incorporated into VLDL in hepatocytes during the assembly of the ApoB lipoprotein particles, our data suggest that dietary LA may cause decreased assembly or secretion of ApoB-coated VLDL. The fact that WD-CO diet concurrently increased cholesterol accumulation in the liver is in support of this possibility. Interestingly, decreased plasma VLDL-TG was not accompanied by elevated hepatic TG accumulation. Such a dissociation may be explained by increased FA oxidation in the liver as reflected by increased production of the ketone body β-hydroxybutyrate.

Two previous studies used whole-body Ldlr knockout (Ldlr-KO) mice to examine the effects of dietary PUFAs against atherosclerosis. In the first study, Merkel and colleagues fed Ldlr-KO mice with experimental diets containing 0.2% wt/wt cholesterol in combination with either coconut oil (SFA) or mixture of corn oil and safflower oil (PUFA)^29^. The mice fed these two diets showed similar plasma VLDL-cholesterol levels and developed equivalent amounts of aortic lesions. In a more recent study, the Ldl-KO mice were fed with either a WD diet enriched in SFA or a WD diet supplemented with conventional soybean oil that predominantly contains LA^30^. Despite its ability to significantly decrease the cholesterol content in VLDL and LDL, the supplementation with soybean oil did not suppress atherosclerotic plaque size when compared to the SFA-rich WD diet. Thus, both studies using the whole body Ldlr KO mice failed to find protective effects of dietary PUFAs against atherosclerosis. This discrepancy from our findings may be due to the fact that the current study used ASO to specifically target Ldlr in the liver. At the established dose of ASO^20^, the expression of Ldlr and thus its contribution in other tissues to lesion development is likely preserved. It is possible that in our model the dietary LA promotes redistribution of LDL-cholesterol from plasma to tissues other than liver, an effect that would be absent in the whole-body Ldlr-KO mice.

A diet rich in linoleic acid (LA) has previously been shown to increase HDL-cholesterol concentration, which was associated with reduced aortic atherosclerotic lesion area in apo-E deficient mice^31^. HDL-cholesterol is involved in the reverse cholesterol transport pathway, thus presence of higher HDL-cholesterol concentration can be expected to have anti-atherogenic effects. However, in the current study, we observed no difference incurred by WD-CO on HDL-cholesterol. We speculate that LA-induced decrease in atherogenic lipid flux may be responsible for lesion reduction in Ldlr-ASO mice treated with WD-CO. Considering that Ldlr was knocked down specifically in the liver, the unchanged HDL-cholesterol suggests that the ability of macrophage foam cells to efflux cholesterol from the growing atherosclerotic plaque is less likely to be affected by the dietary LA.

Our data also showed that consumption of the WD-CO diet not only decreased ApoB secretion, but also stimulated expression of proinflammatory cytokines TNF-α and IL-6 in the liver. LA is a precursor of arachidonic acid that can be converted into proinflammatory eicosanoids such as leukotrienes, prostaglandins, prostacyclins, and thromboxanes^18,32^. Moreover, excessive hepatic uptake of LA may promote lipid peroxidation, and formation of various oxidized forms of LA may directly stimulate inflammation^33^. Thus, our findings are partially in support of this hypothetical “LA-proinflammatory paradigm”. However, despite increased hepatic expression of TNF-α and IL-6, systemic inflammation as revealed by plasma CRP levels appears to be lower in the WD-CO-fed mice. Furthermore, WD-CO feeding over a 12-week period did not induce significant histological effects on liver morphology, inflammation, macrophage content or damage/injury as indicated by plasma AST and ALT levels. It is possible that hepatic inflammation was still at an early stage, during which the beneficial effects of dietary LA on plasma lipids and atherosclerosis outweigh the proinflammatory effects in the liver. In this regard, it would be interesting to determine whether a prolonged WD-CO treatment would maintain its protective effects on atherosclerosis, or it would worsen systemic and tissue-specific inflammation, leading to development of hepatic injury and other metabolic dysfunctions such as insulin resistance.

### Supplementary Information

Supplementary Information.

## Data Availability

The datasets used and/or analyzed during the current study available from the corresponding author on reasonable request.
