# Supplementary material for: Replacement of saturated fatty acids with linoleic acid in western diet attenuates atherosclerosis in a mouse model with inducible ablation of hepatic LDL receptor
Source: Sci Rep. 2023 Oct 6;13:16832. doi: 10.1038/s41598-023-44030-9 (PMC10558454; doi:10.1038/s41598-023-44030-9)

Supplementary Table 1: The FFA composition within the Western diet enriched with butter (WD-B) and Western diet enriched with corn oil (WD-CO).

	Western Diet: Milk Fat (WD-B)		Western Diet: Corn Oil (WD-CO)	
Fatty Acids	*Grams*	% FFA	*Grams*	% FFA
SFA	***122.8***	***62.4%***	***26.9***	***12.9%***
Acetic (2:0)	0	0.0%	0	0.0%
Butyric (4:0)	6.4	3.3%	0	0.0%
Caproic (6:0)	3.8	1.9%	0	0.0%
Caprylic (8:0)	2.2	1.1%	0	0.0%
Capric (10:0)	5	2.5%	0	0.0%
Lauric (12:0)	5.6	2.8%	0	0.0%
Myristic (14:0)	20	10.2%	0	0.0%
Palmitic Acid (16:0)	53.5	27.2%	22.9	11.0%
Stearic Acid (18:0)	24.4	12.4%	4	1.9%
Arachidic (20:0)	1.9	1.0%	0	0.0%
Behenic (22:0)	0	0.0%	0	0.0%
Lignoceric (24:0)	0	0.0%	0	0.0%
MUFA	***60.3***	***30.7%***	***52.3***	***25.1%***
Myristoleic (14:1)	3	1.5%	0	0.0%
Palmitoleic (16:1)	4.6	2.3%	0	0.0%
Oleic (18:1)	52.7	26.8%	52.3	25.1%
Erucic (22:1)	0	0.0%	0	0.0%
PUFA	***13.6***	***6.9%***	***129.2***	***62.0%***
Linoleic (18:2)	10.6	5.4%	126.2	60.6%
α-Linolenic (18:3)	2.9	1.5%	2.9	1.4%
Arachidonic (20:4)	0	0.0%	0	0.0%
Clupanodonic (22:4)	0	0.0%	0	0.0%

Original blots for membranes

Images of original blots presented in manuscript. All membranes were cut prior to assessment with antibodies. Membranes were not cut or modified after labeling with antibodies or imaging the membranes. Cutting the membranes before blotting was done to conserve sample use and reduce off target labeling.

<u>Original blots for membranes presented in Figure 1</u>

Figure 1C: Ldlr

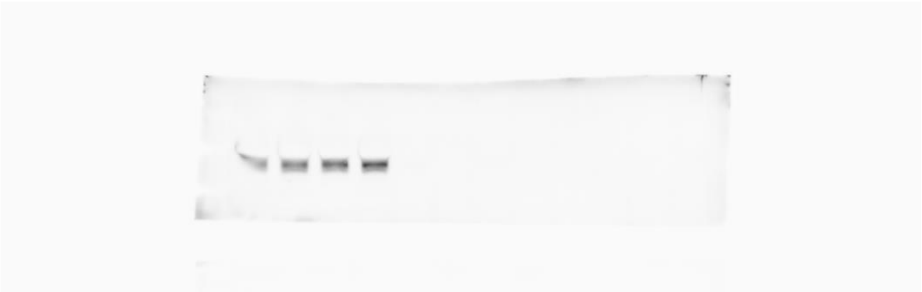

Figure 1C: β-actin

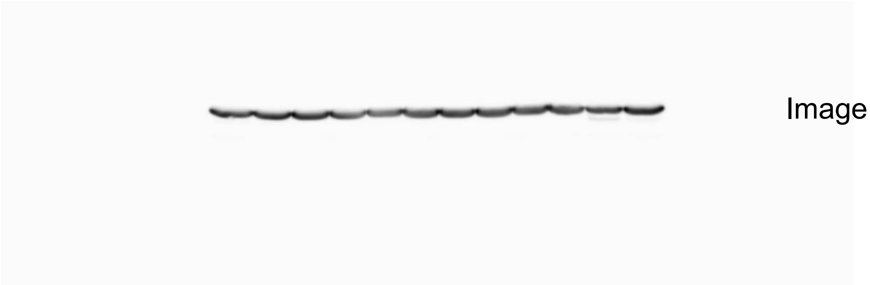

Image that was used in the figure

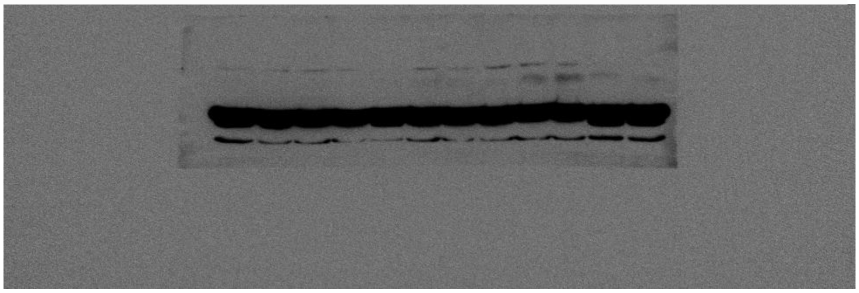

This image was manipulated (enhanced contrast and reduced brightness) for the sole purpose of showing the membrane edges which is a requirement from the journal [illegible]

Ponseau S

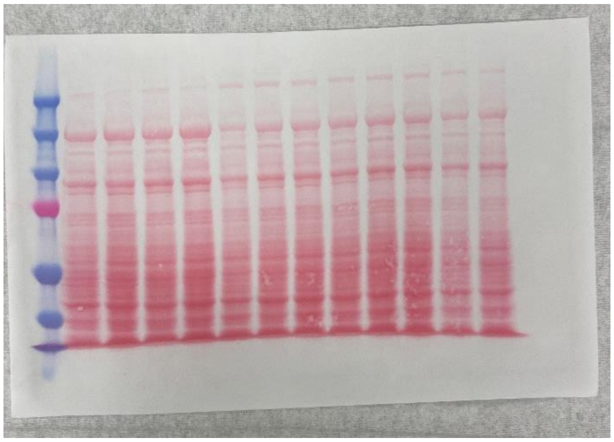

Original blots for membranes presented in Figure 5

Figure 5A: Plasma ApoB

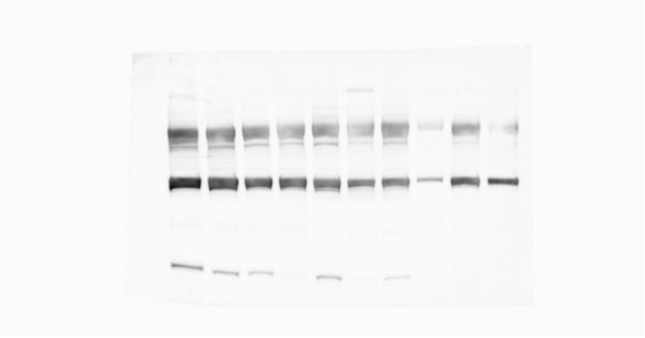

Figure 5A: Plasma Ponseau S

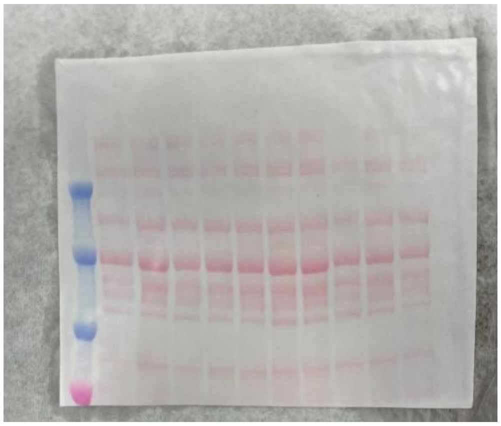

Figure 5B: Hepatic ApoB48

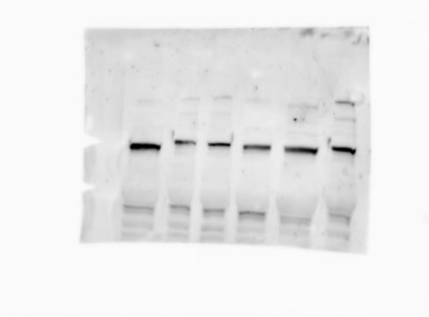

Figures 5B: Hepatic ApoB100

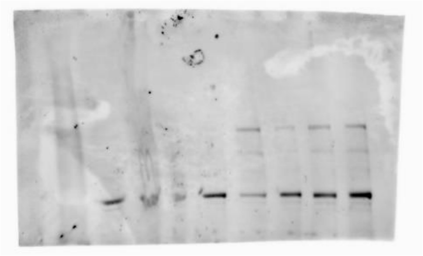

Figure 5B: Hepatic B-Actin

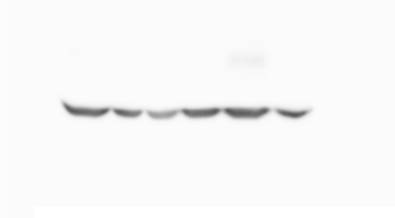

Image used in the figure

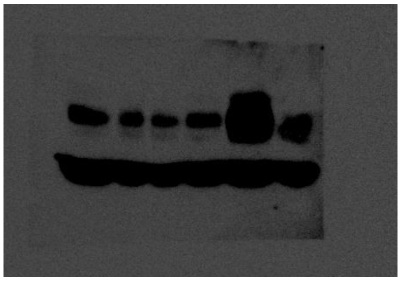

This image was manipulated (enhanced contrast and reduced brightness) for the sole purpose of showing the membrane edges which is a requirement from the journal for publication

Hepatic Ponseau S

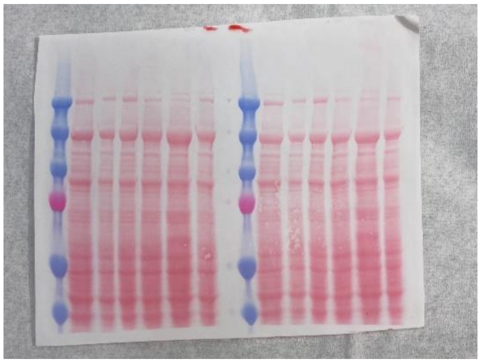

Additional Exposures: These images were requested due to some membrane displaying a darker background. We have included additional images with increasing exposure for transparency.

Additional Exposure for Hepatic ApoB48

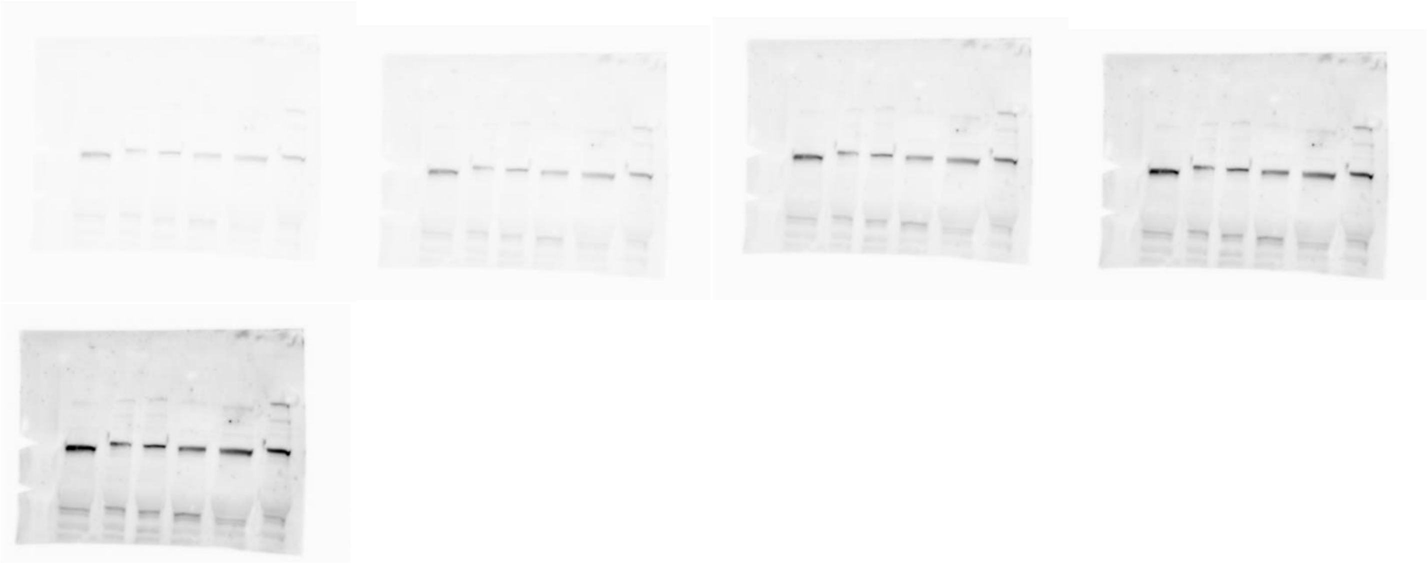

Additional Exposure for Heaptic ApoB100

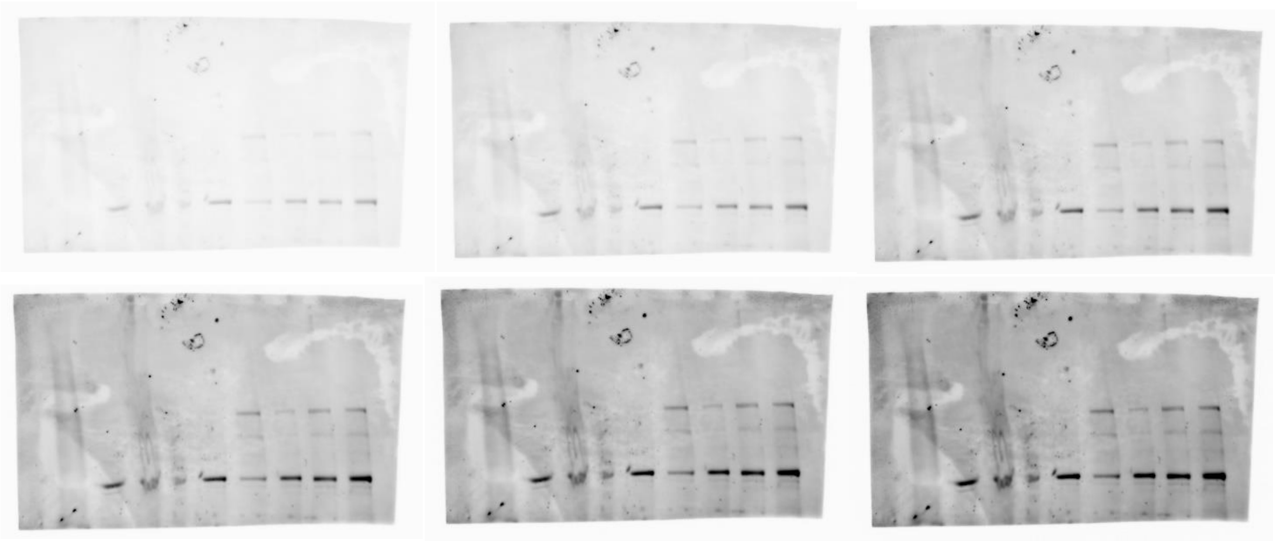

Supplement: Supplementary file 1 — Supplementary Information. [file 41598_2023_44030_MOESM1_ESM.pdf]
